# Dysbiosis and Prematurity: Is There a Role for Probiotics?

**DOI:** 10.3390/nu11061273

**Published:** 2019-06-05

**Authors:** Maria Elisabetta Baldassarre, Antonio Di Mauro, Manuela Capozza, Valentina Rizzo, Federico Schettini, Raffaella Panza, Nicola Laforgia

**Affiliations:** Neonatology and Neonatal Intensive Care Unit, Department of Biomedical Science and Human Oncology, “Aldo Moro” University of Bari, P.zza Giulio Cesare 11, 70124 Bari, Italy; manuela.capozza@uniba.it (M.C.); rizzo.vale.vr@gmail.com (V.R.); federico.schettini@virgilio.it (F.S.); raffaella.panza@uniba.it (R.P.)

**Keywords:** “Dysbiosis” [MeSH], “Premature Birth” [MeSH], “Infant, Premature” [MeSH], “Probiotics” [MeSH]

## Abstract

Healthy microbiota is a critical mediator in maintaining health and it is supposed that dysbiosis could have a role in the pathogenesis of a number of diseases. Evidence supports the hypothesis that maternal dysbiosis could act as a trigger for preterm birth; aberrant colonization of preterm infant gut might have a role in feeding intolerance and pathogenesis of necrotizing enterocolitis. Despite several clinical trials and meta-analyses, it is still not clear if modulation of maternal and neonatal microbiota with probiotic supplementation decreases the risk of preterm birth and its complications.

## 1. Introduction

Modern molecular methods in microbiological diagnostics have recently revealed that modifications in microbial communities in maternal, fetal, and neonatal microbiota play a pivotal role in human health and disease.

In the last decades, dysbiosis has been linked to the origin of many diseases [1]. Evidence supports the hypothesis that maternal dysbiosis could act as a trigger for preterm birth. Furthermore, some aspects usually associated with preterm birth, such as cesarean delivery, formula feeding, and antibiotic exposure, could have an impact on newborn gut colonization and might be partially responsible for feeding intolerance and the pathogenesis of necrotizing enterocolitis.

In this review, we summarize the role of maternal dysbiosis in the pathogenesis of preterm birth, and the role of neonatal dysbiosis in feeding intolerance, necrotizing enterocolitis, and late-onset sepsis. Furthermore, we highlight the new perspectives about microbiota manipulation with prenatal and postnatal probiotic supplementation.

## 2. Methods

An exhaustive search for eligible studies was performed using PubMed, Embase, Medline, Cochrane Library, and Web of Science databases.

The following subject MeSH headings were used: “Premature Birth” [MeSH], “Dysbiosis” [MeSH], “Fetal Membranes, Premature Rupture” [MeSH], “Infant, Premature” [MeSH], “Enterocolitis, Necrotizing” [MeSH], and “Probiotics” [MeSH].

To be as comprehensive as possible, proper Boolean operators such as “AND” and “OR” were also included. Additional studies were sought using references in articles retrieved from searches.

Search limits were set for randomized placebo-controlled clinical trials (RCT), written in English, involving only human subjects, and published between March 2009 and March 2019.

## 3. Vaginal Maternal Dysbiosis and Preterm Birth

Preterm birth, and its complications, are the main cause of death for children below the age of five worldwide. [2] Each year, 15 million babies are born preterm and ascending bacterial infections of the uterine cavity account for the vast majority of spontaneous preterm births.

Rupture of membranes prior to 37 weeks of gestation and before the onset of labor, defined as “preterm prelabor rupture of the membranes” (pPROM), occurs in 30% of spontaneous preterm births [3]. In the majority of pPROM, delivery occurs within 9 days after rupture, and during this time, ascending infections are more likely, leading to an increased risk of chorioamnionitis and funisitis [4].

The clinical management of pPROM is challenging and often controversial: on the one hand, prolongation of pregnancy enables fetal maturation; on the other hand, rupture of the membranes increases risk of infection and poor neonatal outcomes.

The correlation between lower genital tract infection and an increased risk of both pPROM and preterm delivery has lately raised great interest in the pathogenesis of such infection-related mechanisms [5]. However, only a few studies in a small cohort of women with pPROM have been performed after membrane rupture to examine vaginal bacterial composition [6].

During pregnancy, only one or few *Lactobacillus* species dominate the community structure of a healthy vagina [7]. These lactobacilli confer protection against pathobiont colonization by excretion of lactic acid and production of antimicrobial compounds [8].

A recent study that aimed to assess the vaginal microbial community structure using next generation sequencing found an increasing vaginal bacterial diversity with reduced *Lactobacillus* spp. abundance as an early risk factor for pPROM [9].

Using hierarchical clustering analysis of 16S rRNA gene sequencing, Ravel and colleagues classified the vaginal bacterial population into community state types (CSTs) [10]. The CSTs dominated by the presence of bacteria, such as *Gardnerella vaginalis*, *Atopobium vaginae*, and *Veillonellaceae bacterium* were associated with increased risk of early preterm birth. Similarly, an increased presence of *Prevotella*, *Peptoniphilus*, *Streptococcus*, and *Dialister* species was linked with pPROM [11].

Also, bacterial vaginosis might increase the risk of preterm birth and perinatal complications. [12] Despite the mechanisms having not been fully identified, decreased levels of hydrogen peroxide-producing *Lactobacillus* species with altered levels of proinflammatory cytokines are hypothesized to play a pivotal role [13,14].

Despite the fact that human placenta has been traditionally considered as a sterile environment, recent studies revealed that it has a distinct microbiota that can be influenced by maternal conditions, irrespective of the type of delivery [15,16]. Conversely, some authors concluded on the absence of a placental microbiota [17]. However, we can speculate that there is no real reason to determine whether a placental or fetal microbiota exists, as systemic effects of microbial components have been widely demonstrated. [18]

Specifically, microbial species of *Ureaplasma*, *Mycoplasma*, *Aerococcaceae*, Bifidobacteriaceae, and *Fusobacteria* are found predominantly or exclusively in membranes of preterm newborns, highlighting how bacterial ascension from the urogenital tract is the main cause of chorioamnionitis and amniotic inflammation. [9]

Gut and vagina maternal microbiota, via hematogenous spread and ascending colonization, also influence the fetal microbiota composition throughout the pregnancy [19].

Furthermore, increasing evidence suggests that the maternal microbiota, according to its tendency and capacity to gain electrons, influences the placental oxidative reduction potential and might have direct or indirect consequences on the oxidative balance. Hence, gut dysbiosis may both be as the cause as the consequence of increased levels of oxidative species, thus shedding light upon the interplay between the gut microbiota and the placenta in causing congenital malformations and prematurity [20,21].

## 4. Managing Maternal Dysbiosis with Probiotics

It has been argued that probiotics administration during pregnancy and the postnatal period might decrease some maternal and neonatal adverse outcomes [22]. Probiotics are defined by the Food and Agriculture Organization of the United Nations (FAO) as “live microorganisms which when administered in adequate amounts confer a health benefit on the host.” [23]. The most commonly used probiotics include *Bifidobacteria*, lactobacilli, and non-pathogenic fungi.

The probiotics might confer protection against preeclampsia, gestational diabetes, vaginal infections, maternal and infant weight gain, and diseases later in life [22].

Preeclampsia still accounts for a vast number of deaths and severe maternal conditions. It is likely to be the consequence of abnormal placentation and maternal inflammatory response [24]. In this respect, probiotics may have a role in preventing or treating preeclampsia. 

The Norwegian Mother and Child Cohort Study, a large prospective cohort study, revealed an association between consumption and timing of milk-based products with probiotic lactobacilli and decreased risk of preeclampsia [25,26].

Moreover, probiotics can reduce C-reactive protein, a marker of inflammation linked to maternal conditions such as preeclampsia and gestational diabetes [27].

Studies addressing the role of probiotics to improve glycemic status have shown contradictory results. One randomized placebo-controlled study of 256 healthy women treated with diet and probiotics (*Lactobacillus rhamnosus* GG and *Bifidobacterium lactis* Bb12) demonstrated that probiotic supplementation significantly reduced the frequency of gestational diabetes mellitus (13% to 36%) without adverse events in either mothers or newborns [28]. However, a study recruiting 175 obese women demonstrated that supplementation of either a daily probiotic or a placebo capsule from 24 to 28 weeks of gestation did not improve maternal fasting glucose or metabolic profile [29].

Probiotics supplementation may be helpful in bacterial vaginosis as it can restore the vaginal microbiota after antibiotic treatment and decrease the vaginal pH to an optimum value.

Supplementation with probiotics in late pregnancy could modify the vaginal microbiota by counteracting the decrease of *Bifidobacterium* and the increase of *Atopobium*. Moreover, such effects on the vaginal microbiota lead to significant changes in some vaginal cytokines. In particular, the probiotic mixture prevents the decrease of anti-inflammatory cytokines IL-4 and IL-10, and induces the decrease of the pro-inflammatory chemokine eotaxin, thus suggesting a potential anti-inflammatory effect [30]. However, a study by Husain et al. does not confirm such findings, stating that probiotic supplementation is not able to modify the vaginal microbiota [31].

According to a Cochrane meta-analysis, oral probiotics may reduce genital infections by 81%, but data are still insufficient to support a direct effect in preventing preterm labor [32].

A recent systematic review and meta-analysis evaluating the risk of preterm birth and other adverse pregnancy outcomes showed unpromising results. Authors assessed 49 publications representing 27 studies with probiotics supplementation in pregnant women (except for one study that used probiotics). According to their analysis, the administration of probiotics during pregnancy did not affect the risk of preterm birth or other secondary outcomes, including gestational diabetes. The authors concluded that taking probiotics or prebiotics during pregnancy to prevent preterm birth or newborns and maternal adverse pregnancy outcomes is still not supported by evidence [33].

Conversely, probiotics supplementation during pregnancy may help to prevent atopic dermatitis in children. A meta-analysis in 2015 reported data from 4755 children and showed a significantly decreased risk for eczema compared to controls, especially when probiotic supplementation occurred during pregnancy, lactation, and neonatal period. However no significant difference in terms of prevention of asthma, wheezing, or rhinoconjunctivitis was documented [34].

Furthermore, another recent review and meta-analysis showed that probiotics during pregnancy may reduce risk of eczema in children [35].

Supplementation of probiotics to the mothers during pregnancy and/or breastfeeding can also change the cytokine profile of mother’s milk, increase fecal IgA in the offspring [36], and counteract the weight gain of children during the early stages of childhood [37].

## 5. Neonatal Dysbiosis and Consequences for the Premature Infants

The role of neonatal microbial colonization in healthy full-term “vaginally delivered” newborns is a well-known important factor in mucosal immunity, nutrient absorption and digestion, visceral sensing and motility, and finally energy regulation [38]. In healthy full-term newborns, gut colonization is acquired during birth from maternal vaginal and fecal microbiota and, after birth, from breastmilk in breastfed infants [39], and in all infants, including formula-fed ones, from the environment and contacts with parents, siblings, and other relatives [40].

Healthy colonization, called eubiosis, is characterized by a dominance of Gram-positive Firmicutes such as *Staphylococcus*, *Propionibacterium*, *Bifidobacterium*, and *Lactobacillus* with the capacity to digest human milk oligosaccharides. With the transmission of maternal microbiota to the fetus and newborn being a pivotal mechanism in the development of the neonatal gut eubiosis, every factor perturbating this physiological process might be responsible for aberrant gut microbiota colonization, defined as dysbiosis.

The microbiota of children born preterm usually show a prevalence of pathogenic bacteria (e.g., *Klebsiella pneumoniae* and *Clostridium difficile*) with reduced microbial diversity and low levels of short chain fatty acids (SCFA) [40]. Furthermore, compared to term infants, preterm microbiota is dominated by facultative anaerobes, such as Enterobacteriaceae and Enterococcaceae, and low levels of anaerobes such as *Bifidobacterium*, *Bacteroides*, and *Atopobium* [41,42].

This aberrant colonization has been generally linked to absent or limited exposure to maternal microbiota due to shortened labor or cesarean birth [43], exposure to broad-spectrum antibiotics [44], delays in enteral feeding, use of formula feeding [45], and finally to horizontal transmission of bacteria selected in the NICUs’ inhospitable environment. [46]

A recent study showed an increased proportion of Gammaproteobacteria in very low birth weight (VLBW) preterm newborns, with vaginal birth and antenatal steroids identified as a predictor of its abundance [47].

Evidence has shown how preterm infant colonization might be associated with adverse outcomes of prematurity, such as feeding intolerance, necrotizing enterocolitis [48], late-onset sepsis [49], and adverse neurological outcomes later in life [50].

Epithelial structure integrity and barrier function of intestinal enterocyte are influenced by host–microbiota interactions and in preterm infants, the aberrant colonization may be responsible for the structural disruption that increases intestinal permeability, with translocation of bacteria to the bloodstream as well as cell death and necrosis [51].

Preterm immature gastrointestinal tract with a “leaky” barrier and aberrant motility, digestion, and absorption predispose the preterm infant to an increased risk of feeding intolerance [52,53].

Despite feeding intolerance usually represents a benign condition [54], its presentation may largely overlap with that of necrotizing enterocolitis (NEC), which is a severe disease whose pathogenesis is still not fully clarified [55,56]. NEC usually occurs after 2–6 weeks of life, only after microbial colonization of the gut [57,58] that in preterm newborns seems to be markedly influenced by post-menstrual age and gender [59,60]. Recently, systematic reviews and meta-analysis highlighted that an increased abundance of Proteobacteria and a decrease of Firmicutes and Bacteroidetes lead intestinal dysbiosis and precede NEC in preterm infants [61].

In 2019, a case–control study investigated the gut microbial composition of preterm infants with feeding intolerance showing a significantly higher abundance of *Klebsiella* in this population. Despite the fact that no specific microbial pathogen has been identified as being responsible for the development of NEC, authors speculate that *Klebsiella* might be considered as a diagnostic biomarker preceding the onset of feeding intolerance and NEC [62,63].

Late-onset sepsis (LOS) is a major cause of morbidity and mortality in preterm infants due to the immaturity of the immune system in the hostile environment of NICUs [64,65]. Recent papers have investigated the relationship between late-onset sepsis and the microbiota of preterm infants. Despite a causal relationship being not yet certain, data demonstrating that newborns with sepsis showed the same bacteria in their pre-sepsis stool present a strong argument for the hypothesis that aberrant microbial colonization might increase the risk of late-onset sepsis through disruption of the mucosal barrier with secondary translocation of luminal contents [66].

Recently, other available data speculate that neonatal colonization contributes to other short- and long-term sequelae of prematurity, such as respiratory distress and bronchopulmonary dysplasia (BPD), retinopathy of prematurity (ROP) [67], somatic growth in early life, and cardiovascular and other non-communicable chronic diseases later in adulthood [68,69,70].

The role of neonatal microbial colonization of the respiratory tract, in utero and during birth, is a novel research field. Emerging data have highlighted that diverse microbial populations inhabit the respiratory tract, previously considered a sterile compartment [71]. A recent clinical trial examining tracheal aspirates of 71 mechanically ventilated preterm newborns showed important associations between antenatal events, the host airway immune response and airway microbiota [72]. A recent meta-analysis showed that *Ureaplasma* species colonization was significantly associated with the development of BPD, a chronic lung disease of prematurity leading to life-long limitations in lung function [73].

Despite some authors highlighting the possible key role of dysbiosis in the pathogenesis of ROP and non-communicable chronic diseases, this evidence remains somewhat speculative, and other factors, such as respiratory support or diet, need to be further investigated [74,75,76].

Furthermore, evidence supports the hypothesis that intestinal microbiota in preterm infants and the consequent “leaky gut” may alter the systemic inflammatory response. In this view, circulating pro-inflammatory cytokines, oxidative stress, and endotoxemia, resulting in systemic low-grade inflammation and aberrant production of neuroendocrine–immune mediators may exert a direct effect on gut–brain axis with long-term neurodevelopmental impairment [77].

## 6. Managing Neonatal Dysbiosis with Probiotics

The concept of managing the intestinal microbiota or correcting dysbiosis with probiotic supplementation is a promising research field in neonatology [78].

Multiple randomized placebo-controlled clinical trials (RCT) and cohort studies of probiotics in premature infants have been performed.

The main mechanisms for the benefits of probiotics in preterm infants include gut barrier enhancement, immune response modulation, and competitive inhibition of colonization with pathogens.

The differences in microbial colonization between preterm infants and term infants and between preterm infants with NEC and without NEC suggest that interventions focused on modulation of the gut bacteria may play a role in preventing NEC.

In the last decades, different meta-analyses have investigated the efficacy and safety of probiotic supplementation for preventing NEC [79,80,81,82,83,84,85,86,87,88,89].

Despite each meta-analysis having used different inclusion criteria and statistical approach, the evidence is strong regarding the use of combination probiotics of both *Bifidobacterium* and *Lactobacillus* to prevent NEC and reduce mortality in preterm infants of <34 weeks’ gestation or with a birth weight <1500 g [90,91]. Moreover, regarding NEC prevention, the probiotic market raises concerns about the quality and safety of probiotic products, lacking rigorous regulatory oversight [92].

Other positive outcomes of probiotic supplementation seemed to be a shorter time to full enteral feeding [93], shorter hospital length of stay and reduced all-cause mortality. On the other hand, there are still insufficient data on the specific probiotic strain to be used due to significant heterogeneity in clinical trials regarding chosen probiotics and dosing regimens. Thus, more studies are necessary to better understand the influence of prenatal steroids and feeding regimens, and the effect of specific probiotics in high-risk populations such as extremely low birth weight (ELBW) infants.

Two recent systematic reviews and a meta-analysis concluded that probiotic supplementation might reduce the risk of LOS in preterms <37 weeks gestation or with a birth weight <2500 g. The results were also significant in studies that included only infants with gestational age <32 weeks or birth weight <1500 g [94,95]. Further studies are needed to address the optimal probiotic organism, dosing, timing, and duration, and other high-quality and adequately powered trials are warranted to better investigate the efficacy and safety of the use of probiotics in ELBW infants [96]. Furthermore, given the symbiotic properties of human milk, it would be of fundamental importance to understand whether the use of probiotics should be encouraged also in human-milk fed infants, or directed towards exclusively formula-fed infants.

Current evidence also indicates that probiotics can reduce the risk of *Candida* colonization in preterm neonates in NICU [97], despite further high-quality and adequately powered RCTs being warranted [98].

In 2017, a meta-analysis assessing the effect of probiotics on the pathogenesis of ROP demonstrated no significant effect of probiotic supplementation on the risk of developing ROP. Similarly, another meta-analysis assessing the possible role of probiotics in altering the risk of BPD showed no significant effect [99].

Finally, probiotic administration was not beneficial for the prevention of intraventricular hemorrhage (IVH) [85].

Probiotics supplementation is not without risk, particularly in vulnerable populations such as premature infants, where the chance of probiotic translocation and sepsis is higher due to compromised gut integrity. In the literature, cases of probiotic sepsis have been reported [100], and in recent years, concerns have been raised about development and transmission of antibiotic resistance via horizontal gene transfer [101], the possibility of an exaggerated pro-inflammatory response [102], and difficulties in accessing high-quality, safe, and effective products [103,104].

The next frontier for managing dysbiosis in preterm newborns will be the use of post-biotics (metabolites and components from live or dead bacteria) [105] and para-probiotics (non-viable microbial cells, intact or broken, or crude cell extracts) [106], a safe alternative to probiotics as they encompass non-live probiotic products which, when administered in adequate amounts, confer a benefit to the human or animal consumer.

## 7. Conclusions

A healthy microbial colonization (eubiosis) of the maternal vagina and the neonatal gastrointestinal tract seems to correlate with a better newborn state of health.

On the other hand, aberrant or preterm gut microbiota colonization (dysbiosis) might be associated with adverse outcomes of prematurity such as feeding intolerance, necrotizing enterocolitis, and late-onset sepsis. Maternal dysbiosis could represent a risk factor for premature labor as much as preterm newborns dysbiosis could play a role in the state of health and in the pathogenesis of various diseases of prematurity.

Managing dysbiosis and manipulating the microbial environment using probiotic supplementations is a promising research field for promoting health and preventing diseases, reducing the risk of preterm birth and its complications.

In the future, the management of dysbiosis in prematurity could also be based on the use of post-biotics and para-probiotics which, together with probiotics, could provide an additional benefit for premature infants.

Further studies are needed to support these hypotheses and to identify the specific probiotic strain to be used, useful dosage regimens, and the effect of specific probiotics in high-risk populations such as ELBW infants.
